# Impact of passenger engagement through road safety bus stickers in public service vehicles on road traffic crashes in Zambia: a randomized controlled trial

**DOI:** 10.1186/s12889-018-5780-3

**Published:** 2018-07-13

**Authors:** Sydney Chauwa Phiri, Marta R. Prescott, Margaret L. Prust, Elizabeth A. McCarthy, Chuncky C. Kanchele, Prudence Haimbe, Hilda Shakwelele, Sandra Mudhune

**Affiliations:** 1 Clinton Health Access Initiative, PO Box 51071, Ridgeway, Lusaka, Zambia; 2 Clinton Health Access Initiative, Boston, USA; 3 Road Transport and Safety Agency of Zambia, Lusaka, Zambia

**Keywords:** Road traffic crashes, Bus stickers, Insurance claims, Public service vehicle, Injury, Road safety

## Abstract

**Background:**

Road Traffic Crashes (RTCs) are the third highest cause of death in Zambia, claiming about 2000 lives annually, with pedestrians and cyclists being the most vulnerable. Human error accounts for 87.3% of RTCs. Minibus and big bus public service vehicles (PSVs) are among the common vehicle types involved in these crashes. Given the alarmingly high rate of road traffic crashes involving PSV minibuses and big buses within Zambia, there is a need to mitigate this through innovative solutions. In other settings, it has been shown that stickers in PSVs encouraging passengers to speak out against reckless driving can reduce RTCs, but it is unclear whether such an intervention could work in Zambia. Based on this evidence, the Zambia Road Transport and Safety Agency (RTSA) has developed a road safety bus sticker campaign for PSVs and before national scale-up, RTSA is interested in evidence of the impact of these stickers.

**Methods:**

This evaluation will be a stratified two-arm randomized controlled trial with a one-to-one ratio. The sample will be stratified by vehicle type, thus creating a two-arm trial for minibuses and a separate two-arm trial for big buses. The sample will include 2110 minibuses and 300 big buses from four towns in Zambia. The primary outcome of interest will be the difference in the rate of RTCs over a 14-month period (7-months before the intervention and 7 months after) between buses with and without the new RTSA road safety bus stickers.

**Discussion:**

This study will provide evidence on the impact of the Zambian sticker program on road traffic crashes as implemented through minibuses and big buses, that can help inform the scale up of a national ‘Zambia road safety bus sticker campaign’.

**Trial registration:**

PACT-R, PACTR201711002758216. Registered 13 November 2017-Retrospectively registered.

## Background

Globally, road traffic crashes (RTCs) are the 9th leading cause of death and are responsible for about 1.25 million deaths [1]. The burden of mortality from RTCs is disproportionate among certain groups and across regions. Among the youth aged between 15 to 29 years, RTCs are the leading cause of mortality and 90 % of RTC-related deaths occur in low- and middle-income countries despite the fact that these countries account for only 54% of the registered vehicles globally [1]. In particular, Africa has a road traffic fatality rate of 26.6 deaths per 100,000 population, compared to 17.4 deaths per 100,000 globally [1]. Therefore, RTCs have received global attention in recent years, and Sustainable Development Goal targets 3.6 and 11.2 which aim to: by 2020, halve the number of global deaths and injuries from road traffic accidents and by 2030, provide access to safe, affordable, accessible and sustainable transport systems for all, improving road safety, notably by expanding public transport, with special attention to the needs of those in vulnerable situations, women, children, people with disabilities and older people respectively; are specifically targeted at reducing RTCs.

In Zambia, RTCs are the third highest cause of death among the young aged 15–29 years, claiming about 2000 lives annually, with pedestrians and cyclists being the most vulnerable [2]. According to the Road Transport and Safety Agency in Zambia (RTSA),, human error accounts for 87.3% of RTCs in Zambia [3] and the top five human errors include: misjudgment of clearance distance; speeding; failing to keep near the side of the road; cutting in line; and untimely crossing of roads by pedestrians [4]. The most common vehicle types involved in RTCs are public service vehicles (PSVs) and private vans [5]. Though no separate data is available for PSV minibuses, PSV minibuses and private vans combined account for a total of 65% of all RTCs while big PSV buses are second on the list, accounting for 14% of all RTCs [5].

**Fig. 1:**
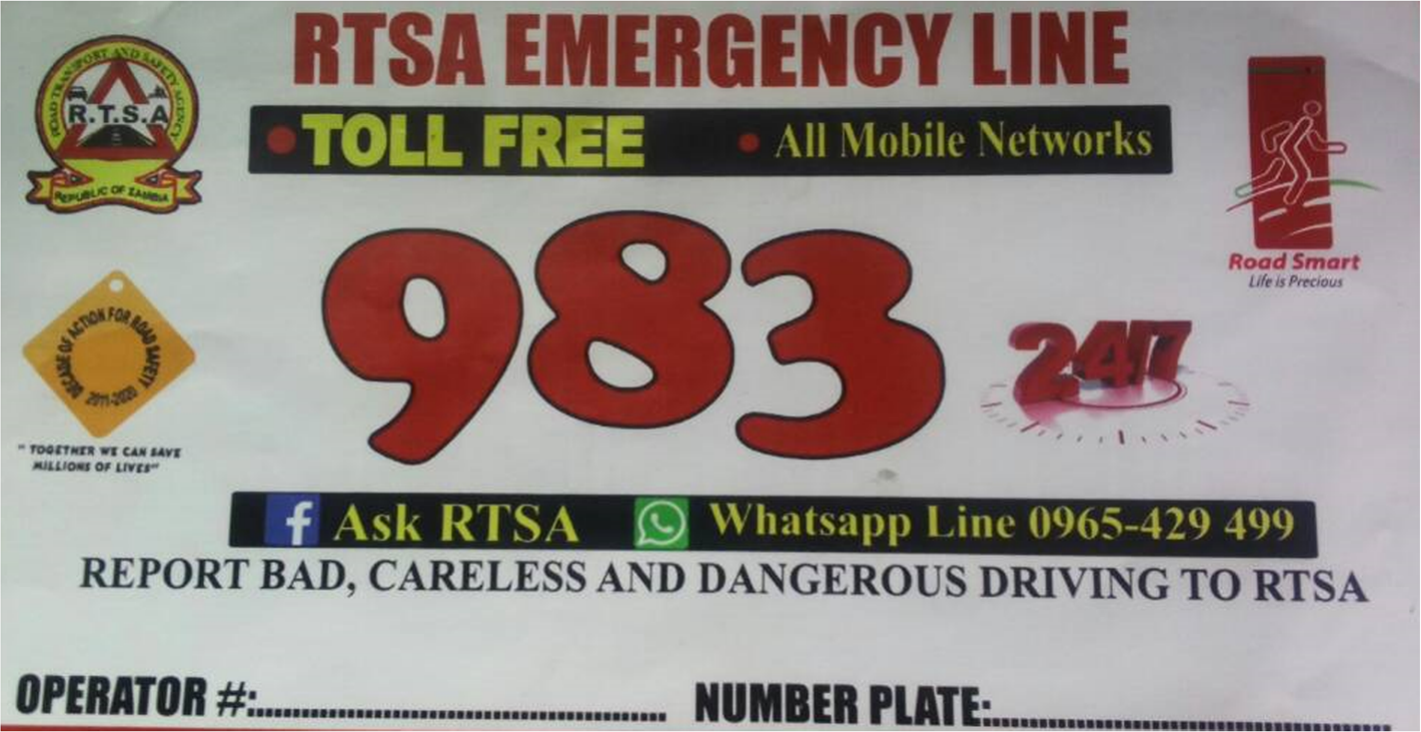
RTSA toll-free bus sticker: This sticker was developed independently by RTSA without input from passengers and drivers of PSV. It provides a toll-free and WhatsApp number to report observed undesirable driving behavior. Rights to publish this image were obtained from the Zambia Road Safety and Transport Agency (RTSA) who developed the image

The RTSA is a quasi-governmental institution mandated to implement and coordinate road safety programs that are aimed at reducing the likelihood and impact of RTCs. Reducing speeding, where speeding is defined as exceeding the speed limit or driving too fast for road conditions, is a key priority for RTSA because of the high rank of speeding among the causes of RTCs and because the measurable nature of this behavior may increase the likelihood that the behavior can be modified. Results from a recent speeding study done by the RTSA in 2016 showed that speeding was most common among PSV minibuses [6]. While it was expected that operators for PSVs would be more careful with speed, the survey found no relationship between the passenger load of the vehicles and their probability of speeding [6]. In an effort to manage speeding or reckless driving in general on Zambian roads and for PSVs in particular, RTSA previously launched several interventions, including road safety bus stickers in July 2016. The original road safety bus sticker provides a toll-free line for passengers to call RTSA and report if the driver is reckless in his driving (Fig. 1). RTSA would in turn call the bus operator/owner, and the operator would then call the driver with that concern.

However, there is a concern that routing feedback from passengers through RTSA and back to bus drivers/ owners may not be efficient in addressing reckless driving and that the messages may not be consistently transmitted. For example, if a passenger called the toll-free number to report a reckless driver and the RTSA call center in-turn tried to call the bus operator /owner who was unreachable at that time, the message would not get back to that driver that there was an incident of reckless driving recorded on that day. In addition, even when the feedback loop is completed from passenger through RTSA to the driver, passengers are still exposed to immediate threat of a road accident due to the long process. Therefore, RTSA decided to consider other sticker designs and messaging to ensure more emphasis is placed on the dangers of reckless driving, and thus potentially prompting feedback to the driver or influencing the driver to drive more carefully.

In the road safety literature, the Haddon matrix is the most common framework used to understand the factors that mitigate injury and death before, during and after a crash [7]. Since majority of traffic crashes in Zambia are caused by human error, the most important human related factors that can be leveraged to prevent crashes according to the Haddon matrix are: information; attitude; impairment and; enforcement [7]. However, much of the evidence on interventions that can prevent road traffic crashes based on the aforementioned human related factors is derived from developed country settings.

In Orlando, Florida evidence suggests that the more passengers a driver carries, the lesser the likelihood of being involved in a road traffic crash [8].This is because the driver’s behavior or attitude would either be self-regulated and/or the passengers would engage the driver if he is driving in an undesirable manner. In Spain, the passing of legislation that increased the likelihood of being jailed for any committed traffic offence significantly reduced the incidence of road traffic crashes [9]. Interestingly, the impact of this legislation intervention had longer lasting effects sustained beyond 3 years [10]. In general however, there are mixed results on the impact of increased law enforcement such as alcohol checks on road traffic crashes depending on the context in which the intervention is evaluated [11–15]. Similarly, there are mixed results on the impact that road safety education programs have on the incidence of road traffic crashes [16–19].

In spite of the dearth of literature on effective interventions that reduce road traffic crashes in developing countries settings, an innovative randomized control trial was conducted in Kenya to test the impact of placing stickers in PSV minibuses encouraging passengers to speak out against dangerous driving [20]. After a period of 8 months, insurance claims rates from PSV minibus road traffic crashes fell by over 50% for minibuses with the stickers compared to those without. Similar studies are now being carried out in Tanzania, Uganda, and Rwanda, and generating context specific evidence would be useful for Zambia as well [21].

### Rationale

Given the alarmingly high rate of road traffic crashes involving PSVs within Zambia, there is a need to mitigate this through multiple measures. One of the most promising interventions is a sticker campaign that was proven effective in Kenya [20]. However, the mixed results on road safety interventions that can reduce road traffic crashes underscore the importance of generating context specific evidence. Therefore, while the problem of reckless PSV drivers exists in Zambia and Kenya, the two contexts are different. Unlike the Kenya study which used weekly financial lotteries to encourage compliance to the intervention, this study proposes to use traffic enforcement officers from RTSA to periodically check for compliance to the intervention. In this regard, the study will implement the intervention within a road traffic law enforcement environment that Zambian PSV drivers are routinely exposed to ensuring that if positive results are seen, they can be easily sustained. Additionally, this new sticker is being introduced into an environment where some passengers have been exposed to a sticker that elicited passengers to report reckless driving to RTSA law enforcement officers as opposed to engaging the driver directly.

### Aim

The aim of this study is to measure the impact of the new RTSA road safety bus stickers in PSVs encouraging passengers to speak out against reckless driving on RTCs involving minibuses. The primary outcome of interest will be the difference in road traffic crashes between buses with and without the new RTSA road safety bus stickers.

## Methods

### Study setting

The evaluation will be conducted as a collaboration between RTSA and the Demand-Driven Evaluations for Decisions (3DE) program of the Clinton Health Access Initiative (CHAI), with support from the Bus Drivers Motor Taxi Association (BDMTA) and the Bus and Taxi Owners Association of Zambia (BTOAZ). RTSA will implement the intervention by placing road safety bus stickers in PSVs. CHAI will lead the evaluation components of the work in cooperation with RTSA. Sample selection will be limited to the populous towns along the portion of the main rail line in Zambia where RTSA enforcement is present, including Kitwe, Lusaka, Livingstone and Kabwe. This selected portion of the main line of rail crosses at least two-thirds of the Zambia’s population and traverses four provinces namely: Lusaka; Copperbelt; Central and Southern provinces. The four towns included in the study are the most populous towns within the respective provinces.

### Designing of new RTSA road safety bus stickers

**Fig. 2:**
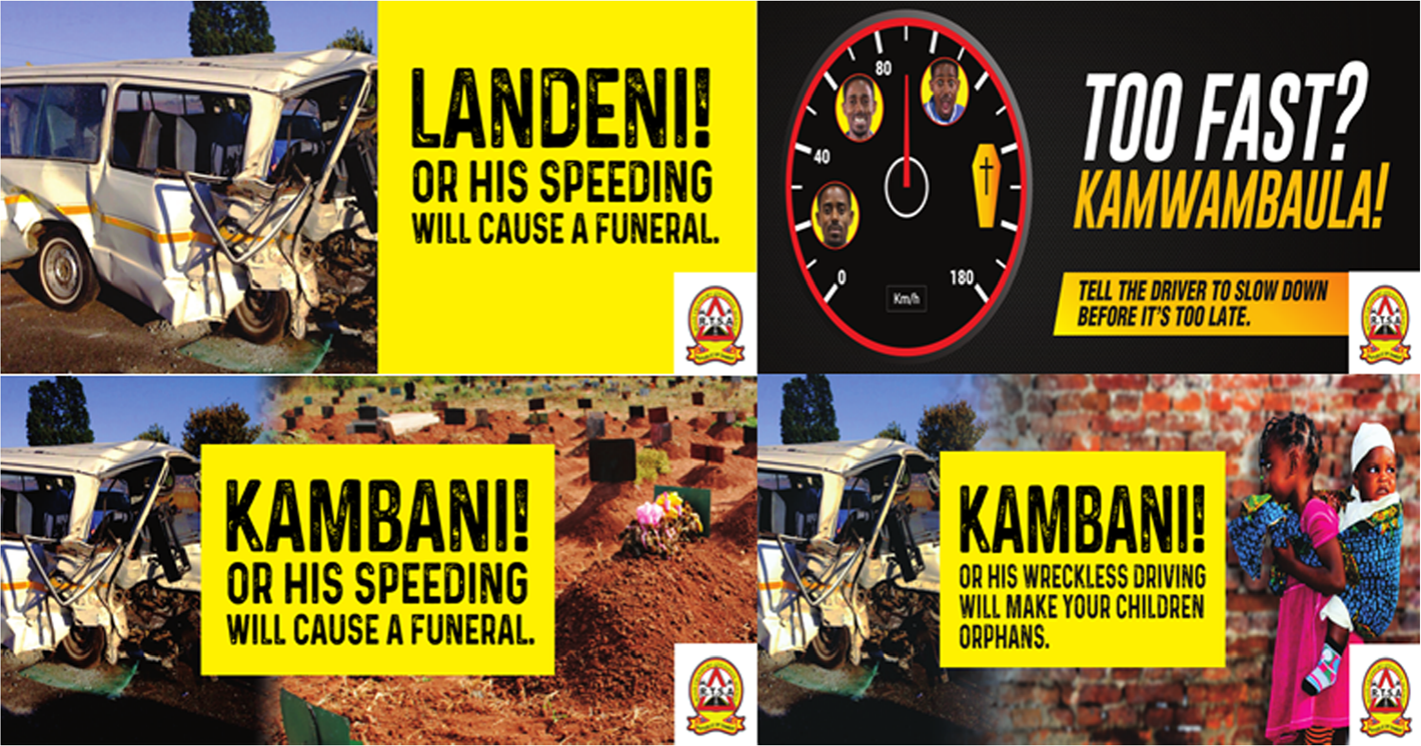
New RTSA road safety bus stickers: Development of this sticker was informed by a series of interviews with passengers and drivers of PSVs. This process led to the development of four themed stickers. Rights to publish these images were obtained from DDB Zambia who developed the images

In order to inform the messaging and design of the new stickers, an informal opinions survey with a mix of open- and closed-ended questions was conducted on a convenient sample of 125 passengers and 40 PSV drivers found at the six main bus stations in Lusaka. This survey obtained opinions on most important considerations that would prompt a passenger to speak out against dangerous driving. Feedback from this informal opinions survey was provided to a creative advertising agency to inform development of the sticker. The design process of the stickers was a four-cycle iterative process of sticker design/redesign and pre-testing of stickers concepts with passengers and drivers. The final road safety bus stickers (Fig. 2) included messaging that had themes of shock, sorrow and speed as these were hypothesized to evoke passengers to speak out. Each of the stickers had a tag line “Speak out” which was translated into the three main languages that are spoken in the sampled towns i.e. “Landeni” (Bemba), “Kambani” (Nyanja) and “Kamwambaula” (Tonga).

### Intervention: New RTSA road safety bus sticker

For the intervention arm of the study, RTSA will be responsible for distribution of the stickers as well as routine inspection of the vehicles to ensure that road safety bus stickers are continuously displayed throughout the evaluation. The stickers will be placed within minibuses and long distance big buses (seating capacity of at least 54 passengers).

In the minibuses, these stickers will be placed on the panels between the passenger window and the ceiling of the vehicle. Approximately six stickers will be placed in each minibus ensuring that at least one sticker is within eye view of each passenger in the vehicle. In the big buses, between 10 and 16 stickers will be placed in each and the placement will vary depending on the design and size of the bus. They could be placed on panels between the passenger window and the ceiling of the vehicle, behind the driver’s seat, or overhead above the televisions, as long as at least one is within eye view of each passenger.

Big buses are routinely inspected before they leave the central bus stations for their long distance journeys. Minibuses will be inspected during routine RTSA road checks, within bus stations and during regular RTSA vehicle inspections. The road safety bus stickers’ campaign and monitoring of minibuses and big buses will occur for approximately 7 months.

### Study design

**Fig. 3:**
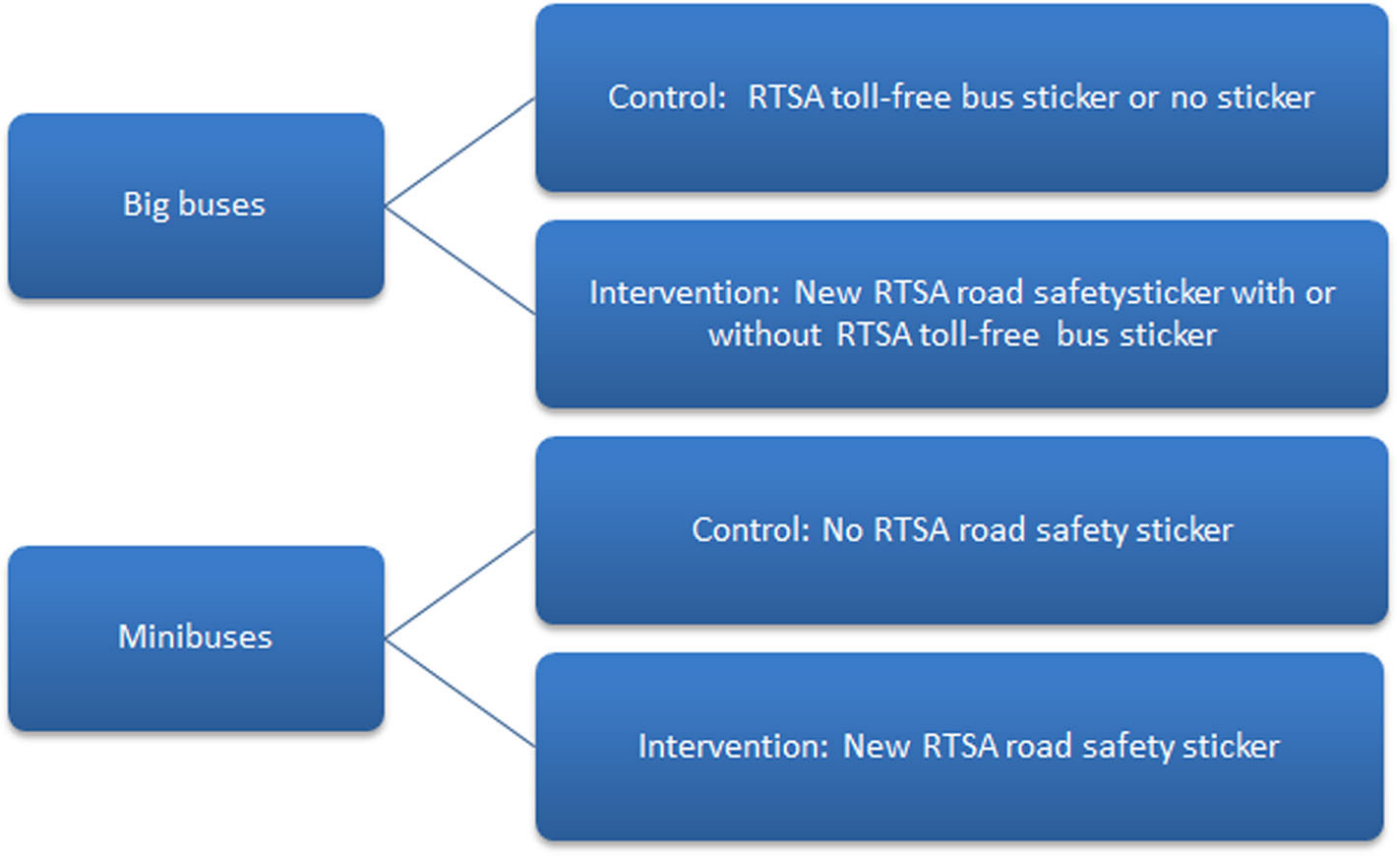
Illustration of study population and stratified two-arm design. The stratification is done by bus type i.e. big buses and minibuses. The big bus two-arm trial differs from the minibus two-arm trial because the RTSA toll-free bus sticker was non-systematically distributed in the big buses while none of the minibuses were exposed to the toll-free sticker

This evaluation will be a stratified two-arm randomized controlled trial with a one-to-one ratio as illustrated in Fig. 3. We will stratify based on vehicle type, thus creating a two-arm trial for minibuses and a separate two-arm trial for big buses.

As the current RTSA toll-free bus stickers have not been distributed among minibuses, the minibus two-arm trial will evaluate the effect of the new RTSA road safety bus sticker over and above no RTSA road safety bus sticker. In comparison, the current RTSA toll-free bus stickers have been non-systematically distributed among all big buses, and thus, the two-arm trial for the big buses will determine the effect of the new RTSA road safety sticker over and above the current system (i.e. combination of RTSA toll-free bus sticker or no sticker). It is hypothesized that the RTSA toll-free bus stickers does not prevent reckless driving, and thus, it will be useful to examine if there is an impact of the new RTSA road safety sticker on big buses with a combination of no road safety bus sticker/toll-free bus sticker.

### Sample size and selection

For a conservative measure of effect, and thus a more conservative and larger sample size, we propose a difference-in-difference primary analysis examining data from 7 months before compared to the 7 months after the implementation of the intervention (and not disaggregated into monthly measures). Based on budget constraints and logistics, we estimated the minimal person time for minibuses assuming we would like to see a minimal effect of 0.01 absolute difference in the *change* in the accident rate per 1000 minibuses over the 14-month period (7-month pre vs. 7-month post). This change is similar to that of the Kenya study. Table 1 presents the inputs used for this equation, assuming the accident rate reported within the Kenya study among the controls would be generalizable to Zambia [20]. To account for potential spillover due to the fact that passengers will transfer between buses (though we assume drivers to remain within the same buses), we assumed that the control arm would have slight improvement in the accident rate over the 14-months observed. Additionally, the equation assumes an alpha of 0.05 and power (beta) of 80%. The equation used is as follows [22]:

$$ N\  person- years\ in\ each\  arm={\left({Z}_{\frac{\alpha }{2}}+{Z}_{\beta}\right)}^2\Big[\left({\lambda}_0+{\lambda}_1\right)/{\left({\lambda}_0-{\lambda}_1\right)}^2 $$

**Table 1:** Sample size calculation parameters

Z_a/2_	Type I error	1.96
Z_B_	Type II error	0.84
*λ*_0_	Estimated change in the accident rate per 1000 minibuses over the 14-month period in the control arm	−0.01
	Absolute difference	.01
*λ*_1_	Estimated change in the accident rate per 1000 minibuses over the 14-month period in the intervention arm	−0.02
y	Number of person years in each arm	116
	Number of cars assuming cars followed for 1.16 years or 14 months	954
	Number of vehicles to follow with 10% buffer per arm	1060

The sample size calculation formula in Table 1 used is adopted from Hayes & Mouton (2009). A buffer of 10% was included to allow for buses that may drop out of the study during recruitment for any reason

For the big buses, there is a finite sample of buses and we intend to enroll all big buses from within the sampled areas.

### Randomization

To randomize the sticker placement, RTSA will place the new RTSA road safety sticker only in buses with license plates ending with an odd number (intervention arm) while buses with a license plate ending with an even number will be considered for the control arm. To reach the suggested 954 per arm, we will aim to enroll approximately 1060 buses per arm, allowing for 10% drop out for any reason.

### Eligibility criteria

#### Inclusion criteria

To be included the PSV must be a licensed operator and must have valid motor vehicle insurance.

#### Exclusion criteria

PSVs will not be included in the study if they do not have valid motor vehicle insurance; and do not have a license to operate as a PSV.

### Study outcomes (Table 2)

ᅟ

**Table 2:** Study Outcomes

No.	Study Comparison	Variable	Indicator	Source
1. Primary Outcome Indicator				
1	Difference between intervention and control buses	Change in traffic crash rate per 1000 buses over a 14-month period	Whether the bus experienced a road traffic crash as reported from motor vehicle insurance claims among those buses during 7-month period of study or 7-months prior to study	Data on motor vehicle insurance claims
2. Secondary Outcome Indicators				
2	Difference between intervention and control buses	Change in the traffic crash rate per 1000 buses overtime for a 14-month period (monthly analysis)	Whether the bus experienced a road traffic crash as reported from motor vehicle insurance claims for each month for the 7-month period of study or 7-months prior to study (repeated measures or monthly analysis)	Data on motor vehicle insurance claims
3	Difference between intervention and control buses	Change in crashes resulting in fatalities per 1000 buses over a 14-month period	Total number of fatalities from road traffic crashes during the period of the study as compared to the 7 months prior to the study	Data on motor vehicle insurance claims
4	Pre vs. Post (overall and not separated per arm)	Mean number of road traffic crashes	Total number of road traffic crashes during the period of the study as compared to the 7 months prior to the study	RTSA data and Zambia road traffic police reports;

Lists one primary outcome of interest and three secondary outcomes of interest. The primary source of information will be data from motor vehicle insurance claims while road traffic police reports will be used for only one secondary outcome

### Study outline (Fig. 4)

ᅟ

**Fig. 4:**
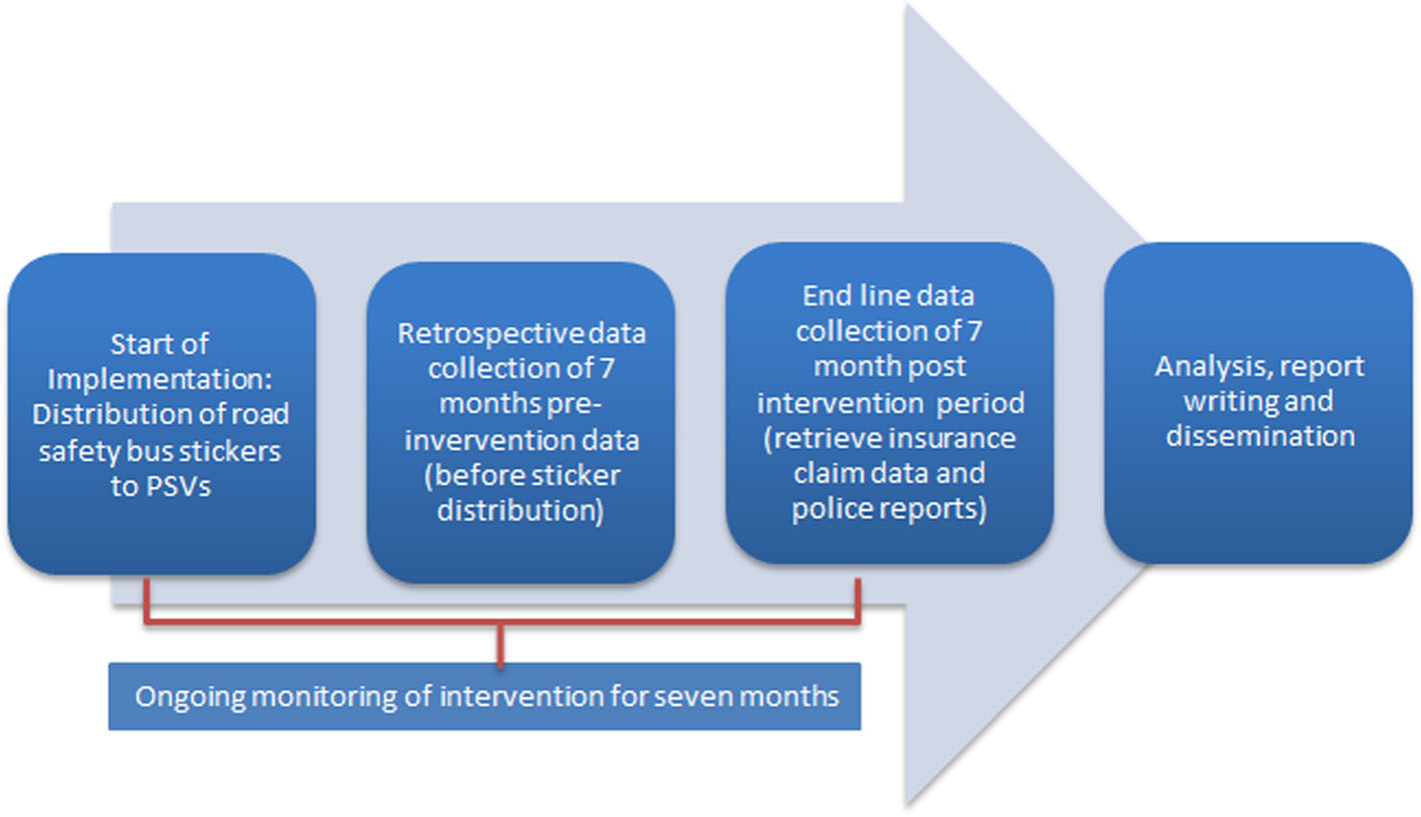
Study outline. The monitoring of the intervention will be done by RTSA as part of their already existing routine inspection exercises of PSVs

### Data collection procedures

#### Motor vehicle insurance claims data

Data on all the claims made during the intervention period will be collected from the general insurance companies where PSVs enrolled in the study have their insurance. The main variables to be reported include date of loss; registration number; location of accident; and classification of accident and severity of accident. An accident will be defined as any collision with another vehicle, pedestrian, animal, or any stationary object such as a tree, pole or building. A claim for the incident does not have to be made with the insurance company for it to qualify as an accident.

#### RTSA data and police reports

As the government body mandated to register and license drivers, motor vehicles and commercial vehicle operators, RTSA maintains records of all PSVs. Any additional data required for analysis will be retrieved from RTSA. In addition, if available and timely, police reports on motor vehicle road traffic crashes for the study period will also be retrieved for analysis.

### Data analysis

At endline, full analysis will be performed using data from the full 14-month period. To assure balance between the study arms, we will compare characteristics between our two arms including insurance company provider and accident rates in the past year. Additionally, utilizing the insurance claims data, we will compare the insurance claim rate per 1000 vehicles in the 7 month period before the intervention.

For the primary outcome of the study in which will examine the change in the road traffic accident rate over the 14-month period for the intervention arm compared to the change in the road traffic accident rate over the 14-month period for the control arm, we will use a difference-in-difference analysis. To this end, we will run a difference-in-difference analysis that will be comparing the intervention and control groups using regression analysis of the following format, and adjusting the SE to account repeated measures (i.e. cluster) from the bus:

$$ {\mathrm{Y}}_{\mathrm{rate}}={\upbeta}_0+{\upbeta}_{\mathrm{Sticker}}{\mathrm{X}}_1+{\upbeta}_{\mathrm{Post}}{\mathrm{X}}_2+{\upbeta}_{\mathrm{Sticker}\mathrm{Post}}{\mathrm{X}}_3+\mathrm{e} $$

For the primary outcome regression, Y is the occurrence of a road traffic crash (indicator 1, 0); X_1_ is the indicator for whether or not the bus was in the intervention arm or control arm (i.e. 0, 1); X_2_ is the indicator for the time period with a value of one indicating the seven-month post intervention period; X_3_ being the interaction indicator between the presence of the new RTSA road safety sticker and the post intervention period (difference-in-difference impact estimate). Additionally in this regression above, the β_Sticker_ estimate will provide the relative difference in the accident rate between intervention and control arm during the pre-intervention period; β_Post_ will be provide the relative change in the accident rate in the control group from pre- to post- intervention period; and, β_StickerPost_ will be the relative difference between the control and intervention group in the change in the outcome from pre to post intervention (i.e. our main estimate of effect). We will appropriately adjust for baseline imbalance between the two arms including insurance provider. To assure outcomes are more meaningful, the rates and thus betas for outcome 1 and 2 will be transformed to report per 1000 minibuses. This regression will then be replicated for the remaining study outcomes.

We will run the difference-in-difference analysis assuming intent-to-treat (ITT), in that all of those who received the assigned new RTSA road safety bus sticker (even vs. odd) will have placed the new RTSA road safety bus sticker accordingly. Though it is expected that the monitoring of RTSA will not provide perfect fidelity to the specific arms, the ITT analysis will provide a conservative estimate of effect.

Due to the nature of the study, drivers and participants cannot be blinded, however, it will be helpful to examine the effect of the new RTSA road safety bus stickers overtime to determine if Hawthorne effects, the process by which a subject of a study changes their behavior due to the knowledge that they are being surveyed and measured, reduces overtime (outcome 2). To this end the regression presented above will become a controlled interrupted time series (ITS) where each minibus will have an observation for each month for the 7-months before the intervention and 7-months after the sticker placement – a form of repeated measures analysis.

In sensitivity analysis, we will examine the difference between the study periods in our primary outcome (seven-month accident rate) controlling for baseline differences.

For outcomes with a comparison over time but not between the two study arms (outcome 4), we will run a general t-test comparison between the RTC rates before and after the intervention as well as a regression analysis controlling for any differences at baseline.

## Discussion

Given the potential infrequency of overall RTCs (0.026 per 1000 minibuses per 6 months as reported in Kenya) and the relatively short length of the study, it is possible we will not find an impact at 7 months. There is a risk that our effect may be conservatively estimated both due to the ITT analysis but also due to potential spillover effects as passengers may go between the arms of the study; however, we feel confident there may still be a potential impact of the new RTSA road safety sticker. This is because the Kenya study similarly randomized sticker placement within the same city, thus allowing for spillover, and was able to see an impact of the sticker program. Additionally, though in the sample size calculation, we accounted for a potential improvement in the accident rate over the 14-months examined in the control group, and estimate we still anticipate some spillover thus resulting in conservative effect estimation.

Though every PSV operator is mandated by law to have valid insurance cover, the use of insurance claims data for our outcome could lead to some measurement error as not everyone files insurance claims when involved in a crash. This may be primarily prevalent in those with third party insurance who may be involved in a crash with unclaimed physical objects such as trees, walk ways, barricades etc. However, RTCs involving injuries or deaths to passengers are highly likely to be reported to insurance companies.

The outcomes, analysis, and recommendations of this evaluation are expected to inform the national scale-up of the new RTSA road safety bus stickers in PSVs by RTSA. Specifically, the results will help RTSA to understand whether the toll-free bus sticker has an impact and whether the new RTSA road safety bus stickers can improve on road safety outcomes. If only limited changes are observed, then RTSA and other national road safety stakeholders will collaborate to understand the reasons and propose further adjustments.

### Dissemination

The outcomes, analysis, and recommendations of this evaluation will be compiled into a study report and policy brief for RTSA. These findings are expected to inform a decision by RTSA about whether or not to scale up the stickers to the national level. If this intervention is scaled across the country, CHAI will support the RTSA in activities such as drafting an operational plan or providing high-level recommendations on how the intervention should be rolled out to achieve optimal results, based on the lessons learned from this evaluation. Pending approval from the RTSA and MOH for wider dissemination, the study results may be published for a wider audience so that these results can influence decision-making in other similar context outside of Zambia.

## Abbreviations

ITS: Interrupted time series analysis
ITT: Intention to Treat Analysis
PSV: Public service vehicle
RTC: Road traffic crash
RTSA: Road Safety and Transport Agency

## References

[1] Organisation, W.H. Global Status Report on Road Safety 2015. Geneva: WHO Press, World Health Organization; 2015.

[2] Zambia, G.o.t.R.o. Report of the Auditor General on Government Measures to Reduce Road Traffic Accidents 2015. Lusaka; 2015. [cited 2017 8 November 2017] . Available from http://www.ago.gov.zm/reports/Special/2015/RATSA.cdr%20final.pdf.

[3] ZAMBIA, N.A.O. Ministerial statement-road traffic accidents in Zambia. 2016 [cited 2017 8 November 2017]; Available from: http://www.parliament.gov.zm/node/5304.

[4] Police, Z. Zambia Police Road Traffic Accidents. 2017 [cited 2017 Nov 6, 2017]; Available from: http://www.zambiapolice.gov.zm/index.php/112-news/321-2017-3rd-quarter-road-traffic-accident-statistics.

[5] Zambia Police Service 2014 Annual Traffic Accident 2015 [cited 2017 12 July ]; Available from: http://zambianroadsafety.org/zambia-police-service-2014-annual-traffic-accident/.

[6] (RTSA), R.T.a.S.A. Speeding Baseline Survey, in Preliminary Report. Zambia: Road Transport and Safety Agency (RTSA), Government Printers, Lusaka; 2016.

[7] Haddon W (1970). On the escape of tigers: an ecologic note. Am J Public Health Nations Health.

[8] Lee C, Abdel-Aty M (2008). Presence of passengers: does it increase or reduce driver's crash potential?. Accid Anal Prev.

[9] Castillo-Manzano JI, Castro-Nuño M, Pedregal DJ (2011). Can fear of going to jail reduce the number of road fatalities? The Spanish experience. J Saf Res.

[10] Izquierdo FA (2011). The endurance of the effects of the penalty point system in Spain three years after. Main influencing factors. Accid Anal Prev.

[11] Erke A, Goldenbeld C, Vaa T (2009). The effects of drink-driving checkpoints on crashes—a meta-analysis. Accid Anal Prev.

[12] Fell JC (2014). Effects of enforcement intensity on alcohol impaired driving crashes. Accid Anal Prev.

[13] Sanem JR (2015). Association between alcohol-impaired driving enforcement-related strategies and alcohol-impaired driving. Accid Anal Prev.

[14] Blais É (2015). Effects of introducing an administrative .05% blood alcohol concentration limit on law enforcement patterns and alcohol-related collisions in Canada. Accid Anal Prev.

[15] Bjørnskau T, Elvik R (1992). Can road traffic law enforcement permanently reduce the number of accidents?. Accid Anal Prev.

[16] Assailly JP (2017). Road safety education: What works?. Patient Educ Couns.

[17] Woratanarat P (2013). Safety riding program and motorcycle-related injuries in Thailand. Accid Anal Prev.

[18] Ivers RQ (2016). Does an on-road motorcycle coaching program reduce crashes in novice riders? A randomised control trial. Accid Anal Prev.

[19] Zeedyk MS (2001). Children and road safety: increasing knowledge does not improve behaviour. Br J Educ Psychol.

[20] Habyarimana J, Jack W (2011). Heckle and chide: results of a randomized road safety intervention in Kenya. J Public Econ.

[21] Georgetown Univesity Initiative on Innovatin, D.a.E. 2017 [cited 2017 12 December ]; Available from: https://gui2de.georgetown.edu/projects/zusha.

[22] Hayes R, Moulton L, Keiding B, Wikle CK, van der Heijden P (2009). Cluster Randomised Trials. Interdisciplinary Statistics Series.

